# Mitigation of apoptosis-mediated neurotoxicity induced by silver nanoparticles via rutaceae nutraceuticals: P53 activation and Bax/Bcl-2 regulation

**DOI:** 10.1016/j.toxrep.2022.11.009

**Published:** 2022-11-29

**Authors:** Rehab M. Abdel-Megeed, Sanaa A. Ali, Wagdy B. Khalil, Esraa A. Refaat, Mai O. Kadry

**Affiliations:** a Therapeutic Chemistry Department, National Research Center, El Buhouth St., Dokki, Cairo 12622, Egypt; b Cell Biology Department, National Research Center, El Buhouth St., Dokki, Cairo 12622, Egypt; c Pharmacognosy Departments, National Research Center, El Buhouth St., Dokki, Cairo 12622, Egypt

**Keywords:** Silver nanoparticle, Neurotoxicity, Bax, Bcl2, P53, Caspase-3, C-reactive protein

## Abstract

Rapid progress in nano-scales and nanostructure extremely altered the way of diagnosing or preventing numerous diseases. One of the most important nano-medicines used in cancer treatment and diagnosis is silver nanoparticles (AgNPs). Regardless of their extensive utilization, their prospective neurotoxicity wasn’t studied yet. Herein, male Swiss Albino mice were intoxicated via two Nano-scales of AgNPs; (20 nm and 100 nm) for one month (100 mg/kg) then treated by leaves extracts of both *Casimiroa edulis* (*C. edulis*) and *Glycosmis pentaphylla* (*G. pentaphylla*), in addition to, mucilage and protein, the separated compounds from *C. edulis* fruits and seeds respectively in a dose of (500 mg/kg). Molecular, Biochemical and histopathological examinations were then conducted. Data recorded showed a significant elevation in hydrogen peroxide (H_2_O_2_) level and reduction in glutathione peroxidase (GPX) level post AgNPs intoxication. The oxidative stress occurred was modulated upon treatment regimens. Protein expression of C-reactive protein (CRP) showed a significant elevation and Molecular analysis recorded a significant up-regulation in the expression of both Bax and caspace-3 genes upon AgNPs intoxication in both particles size. On the contrary, both Bcl2 and P53 gene expression were shown to be significantly reduced. Treatment by *C. edulis*, *G. pentaphylla*, protein and mucilage extracts revealed modulation in apoptotic and pro-apoptotic biomarkers. Histopathological examination confirmed the obtained results. AgNPs exposure could induce neurotoxicity, genetic alternation and oxidative stress; the targeted extracts could be considered as a promising candidate in modulating apoptosis and neurotoxicity induced by AgNPs.

## Highlights

• Silver nanoparticles (AgNPs) are frequently employed in consumer and medicinal products.
• Prolonged use of AgNPs poses harm to human health.
• Investigation of its adverse consequence specifically neurotoxicity is urgent .
• AgNPs intoxication declared an alteration in biochemical and molecular biomarkers.
• Upon natural products treatment, the assessed parameters were modulated.
• Nutraceuticals belong to rutaceae family could modulate neurotoxicity induced by AgNPs.
• It could be promising therapeutic regimen against neurotoxicity induced by AgNPs.

## Graphical Abstract

**Figure:**
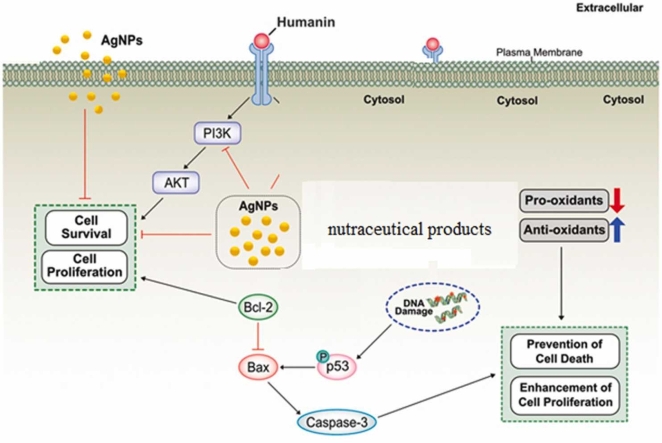

## 1 Introduction

Silver nanoparticles (AgNPs) are frequently employed in consumer and medicinal products, owing to their potent antibacterial and cancer-treating properties. Previous studies reported that prolonged use of AgNPs can harm human health. Therefore, it is urgent to investigate its adverse impact specifically neurotoxicity.

Nanoparticles are active molecules as they can path through cell membrane and interact with intracellular molecules. A former study highlighted the blood-brain barrier (BBB) and brain accumulation of oral AgNPs [1], [2]. Furthermore, passing of AgNPs across the cerebral vessels may affect endothelial cells, alter the cerebral vessels integrity and disrupt tight junction proteins [3], [4], [5].

The mechanisms of toxic effect of nanoparticles especially AgNPs is still unclear despite the high vulnerability of brain tissue to various oxidative stresses. Several in vitro studies investigated AgNPs toxic effects on cellular system that disturb cellular respiration, mitochondrial function and elevate the production of free radical that increase oxidative stress leading to cellular death [6], [7], [8]. Furthermore, animal models declared an obvious oxidative stress upon AgNPs injection based on the alternation of oxidative stress related genes [9], [10], [11].

Previous studies reported that smaller nanoparticles relative surface induced more toxic effects. In regard, it has been reported that 50 and 20 nm AgNPs were less toxic compared with 5 nm particles. Furthermore, smaller sized AgNPs elevated interleukin- 8 gene expression as compared to larger nanoparticles. Furthermore, it has been reported that smaller and larger particles could alter cellular mechanisms leading to cellular apoptosis and then cell death.

Although, AgNps neurotoxicity mechanism is still unclear, alteration in the gene expression, oxidative stress induced by free radical, and apoptosis are the most common underlying mechanisms [12], [13], [14]. Bcl-2 is a protein family which determines cells involvement in apoptosis through mitochondrial interaction. Bcl-2 and other anti-apoptotic biomarkers could inhibit apoptosis, meanwhile Bax as a pro-apoptotic biomarker has the ability to induce apoptosis [15], [16], [17].

Real-time polymerase chain reaction (RT-PCR), one of the most sensitive and responsible methods for analyzing gene expression, was used in the current study to investigate alternations in the expression of the Bcl-2, Bax, and P53 genes in a mouse model following exposure to Ag-NPs.

Various investigations demonstrated plant-derived constituents as antioxidants for treating neurodegenerative disorders [18]. Apoptosis that occurs as a result of neurodegeneration may be prevented via antioxidants via diminishing cellular damage through decreasing loss of neuronal cells progression. Numerous phytochemicals are recorded to have neuroprotective role and are commonly used in traditional medication.

*Casimiroa edulis* (*C. edulis*) is a commonly used plant in wide range as a sedative folk medicine inducing sleep [19]. Numerous studies identified and separated chemical ingredients from the seeds, bark and root of *C. edulis* [20]. Chemical constituents which were separated involved dimethyl histamine and casimiroedine, *N*a, *Na*-dimethyl histamine, imidazole, zapoterin and 2 & 4-quinolinones [21]. *C. edulis* aqueous extract of leaves demonstrated anti-inflammatory as well as diuretic effects. Furthermore, the alcoholic leaves extract recorded anti-mutagenic, anticonvulsant and sedative activities [22].

Numerous studies have supported the use of antioxidants derived from plants in the treatment of neurodegenerative illnesses. By reducing or reversing cellular damage by slowing the loss of neuronal cells, antioxidants may have neurodegenerative effects in addition to their neuroprotective effects (apoptosis prevention). Numerous phytochemicals are known to have neuroprotective effects, but further research is required to understand how they affect the blood-brain barrier. Many plants have diverse biological characteristics and have lately been employed in traditional medicine. *C. edulis* appears promising for its sedative efficiency in inducing sleep [23].

*Glycosmis pentaphylla* (*G. pentaphylla*) is a flowering small plant or evergreen shrub belongs to Rutaceae family. It is mainly found in East as well as Southeast Asia [24]. Numerous active constituents were identified in *G. pentaphylla* such as arborine, flavonoids, terpenoids, alkaloids, coumarins, amides and imides [25], [26].

Diverse parts of *G. pentaphylla* were used in folk medicine in treating various diseases as hook worm infestation, cure boils, ureterolithiasis, chest pain, rheumatism, cough, bleeding and anemia [27], [28], [29]. The juice and leaves were also reported to treat skin affections, eczema, fever, bowel disorder and liver malformation. Moreover, plant wood was traditionally used to treat snakebite in addition to its ability to prevent different types of cancer [30].

*G. pentaphylla*, sometimes known as the gin berry, contains large amounts of phyto-carbazole alkaloids. Numerous pharmacological effects, including anticancer, antioxidant, anti-inflammatory, antibacterial, antifungal, antidiabetic, and neuroprotective activity have been discovered in derivatives of carbazole alkaloids [31].

However, there is no evidence in the prior literature that *C. edulis* or *G. pentaphylla* leaf extract can treat brain damage brought on by AgNPs. In light of this, the main goal of the current study is to investigate the biochemical, histopathological, and molecular changes related to AgNPs induction using high and low particle size in mice model. Furthermore, the therapeutic potential of both *G. pentaphylla* and *C. edulis* leave extracts after neurotoxicity induced by AgNPs was also investigated. The research was also expanded to explore the effectiveness of mucilage and protein, the active components that were extracted from *C. edulis* fruits and seeds respective extract to mitigate the neurotoxic effect induced by AgNPs.

## 2 Materials and methods

### 2.1 Chemicals and reagents

Two particle sized of AgNPs were purchased from Sigma-Aldrich CO. (St Louis, Missouri, USA). Kits for biochemical analysis were provided from biodiagnostic company, Egypt. ELISA Kit for CRP measurement was obtained from (R&D systems, USA). Kits for mRNA extraction, primers specific for Bax, Bcl2 and P53 and one step RT-PCR SYBR green were gained from Qiagen (Helden, Germany). All other used chemicals in the current work are of highly analytical grade.

### 2.2 Used plant

Leaves of *G. pentaphylla* and *C. edulis* were obtained from Mohammed Ali museum and garden botany, Egypt, respectively. Plant taxonomy was identified with Voucher specimens: No: 31–3–2015I & 2015II).

### 2.3 Plant extracts preparation

Briefly, extraction of both *G. pentaphylla* and *C. edulis* were carried out according to the previous published article using air-dried powdered of plant leaves. Firstly powder was defatted in petroleum ether then soaked in methanol at 25 C°. The methanol extract was then evaporated and then, fractionated. Flavonoids were extracted with ethyl acetate at 35 °C under reduced pressure [26].

### 2.4 Preparation of mucilage from *C. edulis* fruits

Five hundred grams of powdered *C. edulis* plant fruits were used to make the mucilage, and the sugar from the hydrolysate's sugar was derivatized and evaluated by GLC under the same circumstances as previously described [22].

### 2.5 Preparation of protein from *C. edulis* seeds

Defatted dried powdered seeds (200 g) were mixed three times (100 ml each) in 10 % sodium chloride solution before being filtered. The experiment was carried out by adding 10 % trichloroacetic acid (TCA) equal volume solution to the filtrate in order to precipitate the protein as a white flocculent amorphous precipitate (2016). Using the previously specified circumstances, the previously synthesized protein (10 mg) was hydrolyzed, and the amino acids of the protein hydrolysate were studied [22].

### 2.6 Design of the experiment

#### 2.6.1 Groupings of animals and treatments

Male Swiss Albino mice (22–25 g/each) were gained from the animal house of our institute (National Research Center, Giza, Egypt). Then they were housed in cages, ten of each allowing standard chow diet and water. According to the institutional animal ethics committee of NRC, the animals used in this investigation received acceptable care and handling [ethical approval No: 16154]. Table 1.

**Table 1:** Forward and reverse primers sequences.

No	Primer Name	Forward primer sequence (5′➙3′)	Reverse primer sequence (5′➙3′)
1	Bax	5′- CTGAGCTGACCTTGGAGC − 3′	5′- GACTCCAGCCACAAAGATG-3′
2	Bcl2	5′- GACAGAAGATCATGCCGTCC-3′	5′- GGTACCAATGGCACTTCAAG-3′
3	P53	5′- CTGTCATCTTCTGTCCCTTC − 3′	5′- TGGAATCAACCCACAGCTGCA − 3′
4	Caspace-3	5′- GGA CCT GTG GAC CTG AAA AA − 3′	5′- GCA TGC CAT ATCATC GTC AG − 3′
5	β-actin	5′- CTTTGATGTCACGCACGATTTC-3′	5′-GGGCCGCTCTAGGCACCAA-3′

One week post acclimatization, animals were randomly subdivided into the following groups (10 /each):

Group 1: Healthy animals.

Groups from group 2–6: mice that were orally administered 20 nm sized of AgNPs in a size of 100 mg/kg for one month [26].

Groups from group 7–11: Animals were administered an oral dose (100 mg/kg) of AgNPs in a size of 100 nm for one month [26].

After that the following regimen was applied:

Groups 2 & 7: Intoxicated animals with AgNPs 20 nm and 100 nm respectively in a dose of (100 mg/kg) and served as positive control [26].

Groups 3 & 8: intoxicated group which treated with *C. edulis* (500 mg/kg) for three weeks; 5 days/week [26].

Groups 4 &9: AgNPs intoxicated mice which were treated with *G. pentaphylla* (500 mg/kg) for three weeks; 5 days/week [26].

Groups 5 &10: AgNPs intoxicated animals which were then administered mucilage (500 mg/kg) for three weeks; 5 days/week [22].

Groups 6 &11: AgNPs intoxicated animals which were then administered protein (500 mg/kg) for three weeks; 5 days/week [22].

At the end of the experiment, blood samples were collected from the sublingual vein. Sera were separated by centrifugation at 4000 rpm for 15 min and were kept at − 80 °C for subsequent assessment of biochemical parameters.

Mice were then euthanized by Co_2_ asphyxiation. After that, brain tissue was carefully separated for molecular and histopathological examination and kept in − 80 °C and 10 % formaldehyde respectively.

### 2.7 Estimation of antioxidants

Antioxidant activity was estimated for all groups. Hydrogen peroxide (H_2_O_2_) and Glutathione Peroxidase (GPx) were estimated according to Aebi, 1984 [32] and Paglia & Valentine, 1967 [33], respectively.

### 2.8 Measurement of the inflammatory CRP biomarker

C-reactive protein (CRP) was measured in serum samples using ELISA technique according to the instructions of manufacturer then read the absorbance in spectrophotometer at 450 nm [34].

### 2.9 RNA extraction and RT-PCR analysis for apoptosis determination

Total RNA was isolated from the brain tissue using Qiqamp mini kit (Qiagen; USA) according to the manufacturer’s instructions. Then subjected to RT-PCR analysis to quantify mRNA gene expression of Bax, Bcl2, P53 and caspases-3 using one step QuantiTecht SYBR green (Qiagen; USA) RT-PCR reaction was performed according to our previously published article [35]. Suitable annealing temperature was determined for each primer. The relative expression for each studied gene was gained by comparative CT (2-^ΔΔCT^) methodology [35].

### 2.10 Histopathology

Brain tissues were cut into representative slices, which were subsequently fixed in 10% formalin buffer. After fixation, 4-mm thick paraffin-embedded slices were obtained, and hematoxylin and eosin (H&E) slide staining was done to analyze each sample under a light microscope [36].

### 2.11 Statistical analysis

Version 16 of SPSS software program was used to analyze the obtained data. The mean ± SE was used to express all values. Turkey's multiple comparisons post hoc test was used to statistically examine significant differences between the groups after one-way analysis of variance (ANOVA). Significant differences were defined as those with p.05.

## 3 Results

### 3.1 Characterization studies

**Fig. 1:**
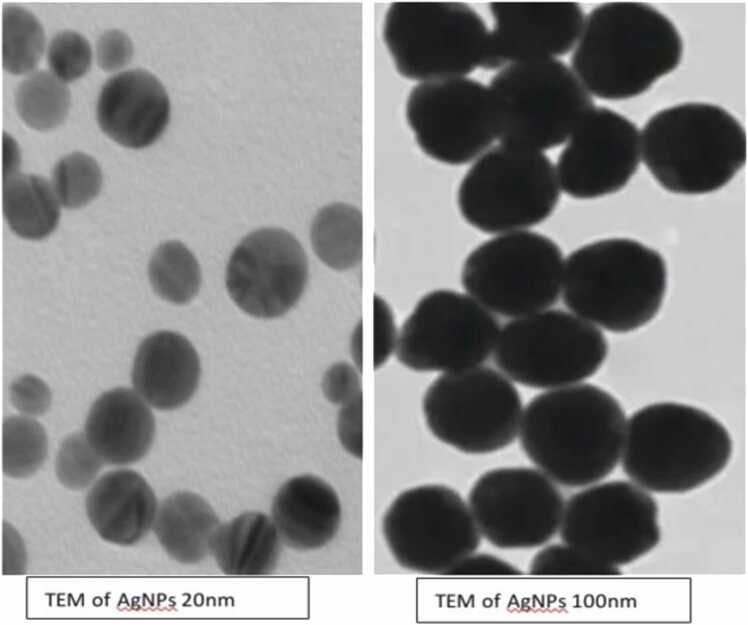
Transmission electron microscopy (TEM) of both 20 nm and 100 nm AgNPs.

The size of the two used nanoparticle was elucidated using transmission electron microscopy (TEM) was 20 ± 3 nm and 100 ± 11 nm recording a hydrodynamic diameter potential (Fig. 1).

### 3.2 Oxidative stress modulation

**Fig. 2:**
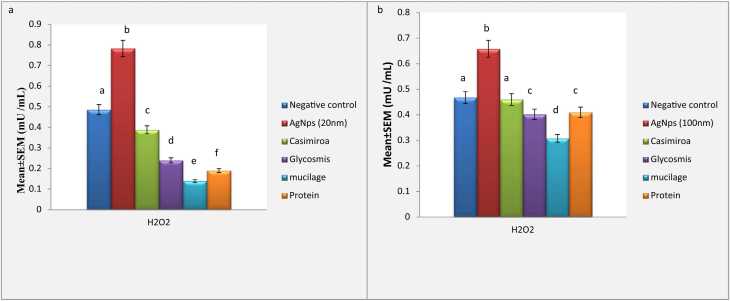
**a:**, Effect of AgNps (20 nm) intoxicated rats on hydrogen peroxide (H2O2). Data are presented as means ±SEM. Relatively to healthy groups, unlike letters are deemed significant*.* P < 0.05. **b:**, Effect of AgNps (100 nm) intoxicated rats on hydrogen peroxide (H2O2). Data are presented as means ±SEM. Relatively to healthy groups, unlike letters are deemed significant*.* P < 0.05.

**Fig. 3:**
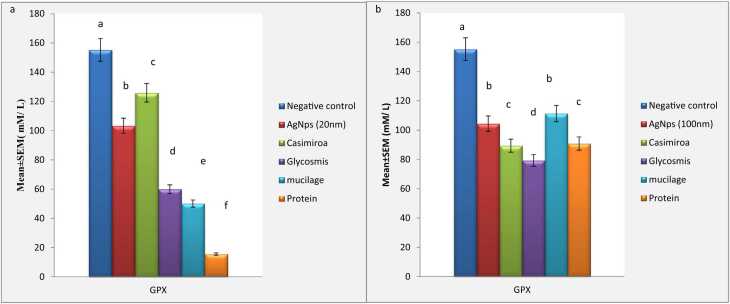
**a:**, Effect of AgNps (20 nm) intoxicated mice on Glutathione Peroxidase (GPX). Data are presented as means ± SEM. Relatively to healthy groups, unlike letters are deemed significant*.* P < 0.05. **3b:**, Effect of AgNps (100 nm) intoxicated mice on Glutathione peroxidase (GPX). Data are presented as means ± SEM. Relatively to healthy groups, unlike letters are deemed significant*.* P < 0.05.

Silver nanoparticles-intoxicated mice significantly elevated H_2_O_2_ levels besides an obvious relief was recorded in GPX values against the control group (Fig. 2 & Fig. 3). *C. edulis* treatment improved significantly of the two sized AgNPs intoxicated groups. Meanwhile, *G. pentaphylla*, Mucilage and Protein treatment after 100 nm AgNPs intoxication declared a significant modulation of oxidative stress biomarker H_2_O_2_ biomarker to be near the normal value. Meanwhile, a non-significant improvement was observed in groups treated by *G. pentaphylla*, Mucilage and Protein after 20 nm AgNPs intoxication.

### 3.3 CRP Inflammatory biomarker modulation

The data revealed in the current work recorded a significant elevation in CRP values upon 20 nm Ag-NPs intoxication as well as 100 nm with more toxic effect than 100 nm. Whereas, all treated groups declared a significant modulation in CRP levels with the excellence of *G. pentaphylla* treatment over all the other treated groups as compared positive control groups at p < 0.05 (Table 2).

**Table 2:** Effect of *C. edulis, G. pentaphylla*, mucilage and Protein on serum C-reactive protein post Ag NPs (20 & 100 nm) intoxication. Data are, expressed as means ± SEM (n = 10). p-value < 0.05 is considered significant. Groups having the same letter are not significantly different, while those having different letters are significantly different from each other.

Groups	Ag-NPs (20 nm) M ± SE	Ag-NPs (100 nm M ± SE
Negative control	4^**a**^ ± 0.6	4^**a**^ ± 0.6
Positive control (Ag-NPs)	100^**b**^ ± 2.5	90^**b**^ ± 3.3
*C. edulis*	45^**c**^ ± 1.7	25^**c**^ ± 1.9
*G. pentaphylla*	40^**c**^ ± 2.4	5^**a**^ ± 0.3
mucilage	65^**d**^ ± 2.8	56 ^**f**^ ± 3.2
Protein	81^**e**^ ± 4.1	69 ^**g**^ ± 3.6

### 3.4 Modulation of Bax/Bcl2 gene expression

Silver nanoparticles intoxicated group declared a significantly elevated Bax gene expression by fold change 2.1 and 1.78 for both 20 nm and 100 nm of silver nanoparticles, respectively against negative control. Meanwhile, a significant reduction was observed in Bcl2 gene expression upon 20 nm silver nanoparticles intoxication (0.5 fold change). Whereas intoxication by particle size 100 nm declared a non-significant reduction in Bcl2 gene expression (0.98 fold change).

Furthermore, *C. edulis* treatment declared a significant modulation of Bax gene expression in 20 nm intoxicated group (1.09 fold changes) as well as 100 nm intoxicated group (1.2 fold change) to be near the normal value. In addition, a significant reduction in Bax gene expression was observed upon *G. pentaphylla* treatment (1.1 & 1.54 fold changes for 20 nm and 100 nm respectively). Upon *G. pentaphylla* treatment, Bcl2 gene expression was elevated to be near the normal healthy group in 20 nm intoxicated group (0.91 fold change). In contrast, a non-significant elevation was declared in 100 nm intoxicated groups (0.86 fold change).

Treatment by mucilage extract declared significant reduction of Bax gene expression in 100 nm intoxicated group (1.51 fold change). Otherwise, non-significant reduction was recorded in the expression of Bax gene to be near the normal value (2.05 fold change).

Mucilage 100 nm intoxicated group treatment could reduce Bcl2 gene expression but not statistically significant (0.78 fold change). Moreover, a significant reduction was declared in the expression of Bcl2 gene to be near the normal value (0.8 fold change).

**Fig. 4:**
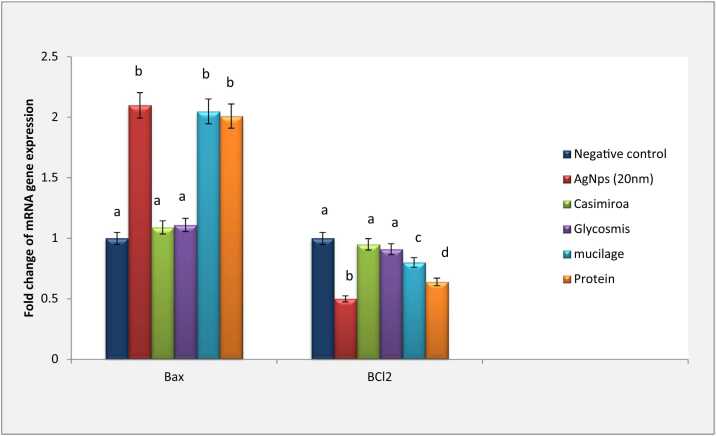
Effect of AgNps (20 nm) intoxicated mice on mRNA gene expression of both Bax and BCl2. Data are presented as means ± SEM. Relatively to healthy groups, unlike letters are deemed significant*.* P < 0.05.

**Fig. 5:**
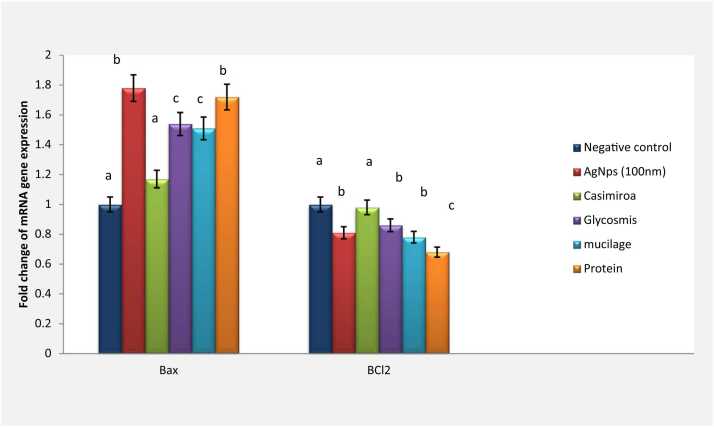
Effect of AgNps (100 nm) intoxicated mice on mRNA gene expression of both Bax and BCl2. Data are presented as means ± SEM. Relatively to healthy groups, unlike letters are deemed significant*.* P < 0.05.

Protein extract treatment declared non-significant reduction in Bax gene expression in both intoxicated silver nanoparticles sized (1.72 & 2.1 fold changes). Bcl2 gene expression declared a significant reduction in 20 nm as well as 100 nm intoxicated groups (Fig. 4 & 5).

### 3.5 Modulation of over-expression of P53 induced by silver nanoparticles

Data recorded in the present research declared an obvious elevation in P53 gene expression induced by silver nanoparticles intoxication in both 20 nm and 100 nm (1.7 &1.8 respectively).

**Fig. 6:**
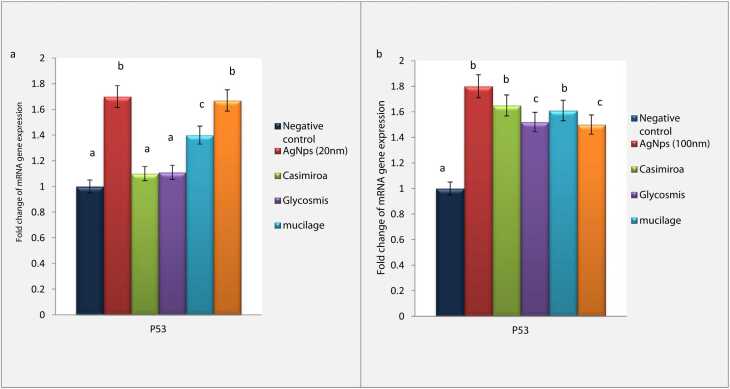
(a) Effect of AgNps (20 nm) intoxicated mice on mRNA gene expression of P53. Data are presented as means ± SEM. Relatively to healthy groups, unlike letters are deemed significant*.* P < 0.05. (b)**:** Effect of AgNps (100 nm) intoxicated mice on mRNA gene expression of P53. Data are presented as means ± SEM. Relatively to healthy groups, unlike letters are deemed significant*.* P < 0.05.

Treatment by *C. edulis* declared a significant modulation of P53 gene expression in 20 nm intoxicated group (1.1 fold change) whereas non-significant improvement was declared in 100 nm intoxicated group upon *C. edulis* treatment (1.65 fold change). In addition, a significant reduction in P53 gene expression was observed upon *G. pentaphylla* treatment (1.11 & 1.52 fold changes for 20 nm and 100 nm respectively). Mucilage extract treatment showed an obvious reduction of the expression of P53 in 20 nm intoxicated group (1.4 fold change). Otherwise, non-significant reduction in the expression of P53 was declared in 100 nm intoxicated group upon mucilage treatment (1.61 fold change). Upon protein administration, P53 gene expression was reduced significantly in 100 nm intoxicated group (1.5 fold change) further, non-significant reduction was observed in P53 gene expression in 20 nm intoxicated group that treated by protein extract (1.67 fold change) (Fig. 6a & 6b).

### 3.6 Modulation of caspace-3 biomarker

The current study investigated a remarkable elevation in the expression of caspace-3 induced by silver nanoparticles intoxication in both 20 nm and 100 nm (2.67 &3.22 respectively).

**Fig. 7:**
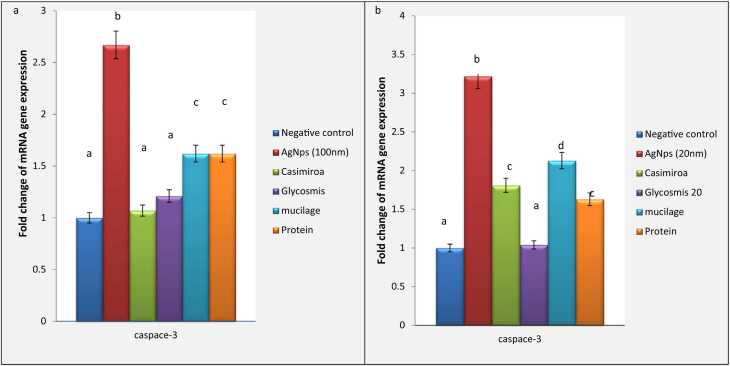
(a) Effect of AgNps (20 nm) intoxicated mice on mRNA gene expression of caspace-3. Data are presented as means ± SEM. Relatively to healthy groups, unlike letters are deemed significant*.* P < 0.05. (b)**:**, Effect of AgNps (100 nm) intoxicated mice on mRNA gene expression of caspace-3. Data are presented as means ± SEM. Relatively to healthy groups, unlike letters are deemed significant*.* P < 0.05.

Treatment by *C. edulis* declared a significant downregulation in the expression of caspace-3 in 20 nm intoxicated group (1.07 fold change) to be near the normal value. Furthermore, a significant improvement was declared in 100 nm intoxicated group upon *C. edulis* treatment (1.81 fold change). Upon *G. pentaphylla* treatment, a significant reduction in the expression of caspace-3 was recorded of 20 nm as well as 100 nm groups (1.2 & 1.04 respectively). Mucilage extract treatment declared more significant improvement in the expression of caspace-3 in 20 nm intoxicated group (1.62 fold change) than those in 100 nm (2.13 fold change). Otherwise, non-significant reduction was observed in the expression of caspace-3 in 100 nm intoxicated group upon mucilage extract (1.61 fold change). Meanwhile, protein extract treatment was significantly reduced in 100 nm intoxicated group (1.5 fold change). Otherwise, non-significant reduction was recorded in the expression of caspace-3 in both 20 nm and 100 nm intoxicated groups (Fig. 7a & 7b).

### 3.7 Histopathological examination

**Fig. 8:**
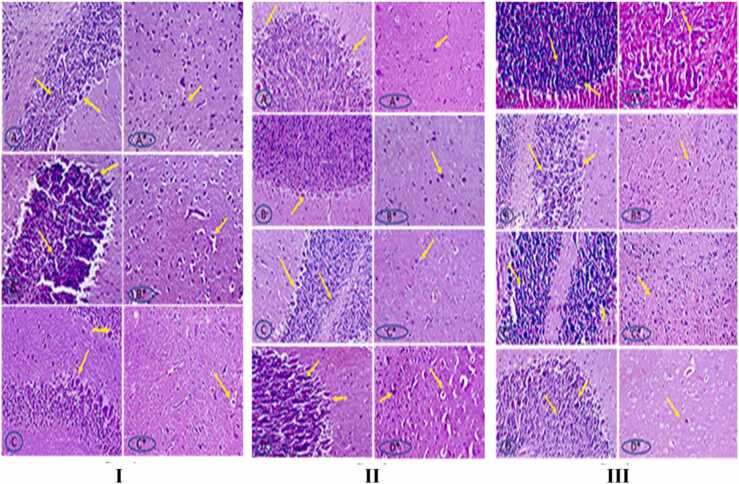
I (A&A*): Brain section of control mice, showed normal histological features. (B&B*): Brain section of +ve 20 nm group (C&C*): Brain section of +ve 100 nm group. II (A&A*): Brain section of 20 nm treated *C. edulis* group, the cerebellum showed almost normal histological features. (B&B*): Brain section of 20 nm treated *G. pentaphylla* group. (C&C*): Brain section of 20 mm treated mucilage group (D&D*): Brain section of 20 mm treated protein group. III (A&A*): Brain section of 100 nm treated *C. edulis* group (B&B*): Brain section of 100 nm treated *G. pentaphylla* group. (C&C*): Brain section of 100 nm treated mucilage group (D&D*): Brain section of 100 nm treated protein group; (H&E stain 200, ×400).

According to Fig. 8 and section I The control group's brain tissue displayed typical histological characteristics, including well-defined molecular and granular features and normal-looking neuronal cell structures: The frontal cortex of the +ve 20 nm group had severe structural disarray, edoema, and somewhat pyknotic cells, while the cerebellum had mildly smaller cells overall. The frontal cortex of the +ve 100 nm group's brain part displayed severe structural disarray and edoema, whereas the cerebellum displayed thinness and a reduction in cellular size. Additionally in part II: The cerebellum from the treated *C. edulis* and *G. pentaphylla* groups displayed nearly normal histological characteristics, illuminating a well-defined molecular granular structure. The cerebellum from the treated mucilage group displayed almost normal histological characteristics. The treated protein group revealed nearly normal histological changes in the cerebellum.

## 4 Discussion

A pressing need to understand the neurotoxic impact of AgNPs, regarding their wide applications and great ability to cross the BBB and then tend to accumulate in the brain upon exposure [37]. Previous investigations proved that AgNPs doesn’t only accumulate in the brain but also induce particular degree of tissue damage [38]. Therefore, there is an urgent need to declare the possible action of AgNPs on brain tissue.

In the current work, experimental animals were intoxicated by two different sizes of AgNPs. The results demonstrated that AgNPs induced dysfunction of brain tissue in addition to different oxidative stress that induced alternation on various biomarkers. Neurotoxic impact of AgNPs was illustrated in several studies utilizing different particle sizes [13], [17]. Exposure to AgNPs, can cause toxicity in different organs, inclusive the brain and nervous system [39], [40], [41]. The biological half-life of Ag ions in the central nervous system is longer than that in other tissue organs, indicating that it might cause significant neuro-physiological dysfunction and may cause a risk to the brain [4]. Accumulation of AgNPs in different regions of the brain tissue can cause their damage [41].

Possible mechanism that is responsible for AgNPs toxicity includes oxidative stress as well as genotoxicity that finally ends in cell apoptosis [42]. Oxidative stress associated with AgNPs neurotoxicity is mediated via the influence of reactive-oxygen species, exhaustion of glutathione values and increment in caspase-3 level [43]. Furthermore, over-production of ROS induces neurotoxicity in neurons through alteration in the genes expression which are responsible for oxidative stress [44].

Data in this work revealed a significant increment in H_2_O_2_ levels on the other hand, a significant reduction in GPX values against negative control value was observed. Meanwhile, *C. edulis* treatment declared a significant modulation in oxidative stress biomarkers in the two particle sizes. However, 100 nm AgNPs intoxication followed by *G. pentaphylla*, Mucilage and Protein treatment demonstrated a remarkable improvement in oxidative stress biomarkers. Meanwhile, a non-significant improvement was declared upon *G. pentaphylla*, Mucilage and Protein treatment of 20 nm AgNPs groups.

However, an elevated CRP levels was oblivious upon AgNPs intoxication, its value was significantly reduced in all treated groups relative to negative control value. AgNPs can induce toxic effect and trigger various tissue responses as generation of reactive oxygen species, inflammation, and finally cell death [45], [46]. CRP elevated values suggest the existence of reactive species regarding the prevalence of numerous pathological conditions such as inflammation. However, treatment by the above mentioned plant extracts can effectively reduce oxidative damage through reactive oxygen radicals scavenging property and their antioxidant effect [47].

In the current study, AgNPs intoxicated group declared a significant reduction in Bcl-2 and P53 gene expression beside an obvious increment in the pro-apoptotic Bax gene expression. The current result confirmed that the apoptosis cascade contributes to the cytotoxicity related to AgNPs. These findings are in the agreement with earlier studies which showed regulation of these genes from cell lines of human liver (HEPG-2), human colon adenocarcinoma, and hamster kidney upon AgNPs intoxication [48], [49]. Meanwhile, AgNPs responsible for Bcl-2 stimulation, is regulated by P53 over-expression [48]. Mott et al. suggested that ROS are signaling molecules which initiate and execute apoptotic cell death cascade [50]. Furthermore, ROS production is also responsible for apoptosis in numerous situations such as neurodegeneration and inflammation [51]. Moreover, upregulation of both mRNA and protein levels of Bax was recorded whereas a significant downregulation was declared in the level of Bcl2 mRNA expression in human neuronal cells treated with Gadolinium oxide nanoparticles [52].

The binding of Bax to mitochondrial membrane leads to p53-mediated apoptosis. Then caspases are activated in numerous cells playing an essential role in the initiation as well as the accomplishment of apoptosis. The activated caspase-3 was previously reported to be necessary in DNA damage and apoptosis [53]. On the other hand, overexpression of p53 stimulates pro-apoptotic Bcl-2 family such as Bax and increase permeability of mitochondrial membrane leading to the release of some proteins into the cytosol, where they activate caspase-9 which further activates caspase-3 (the effector caspase) which are important enzymes in apoptosis [52], [54]. In the present study, the RT-PCR results can be considered as good confirmatory biomarker for apoptosis upon AgNps neurotoxicity. Furthermore, an obvious improvement was found after treatment via these natural products.

In the current study, treatment by *C. edulis* leaves extract, *G. pentaphylla,* mucilage and protein declared a significant mitigation in all biochemical, oxidative as well as molecular measured parameters. These findings suggested that the above mentioned extracts possess neuroprotective, and anti-apoptotic potential against AgNPs induced cerebral damages and can induce beneficial therapeutic effects by suppressing the process mediated with free radical formation; an action that could be related to the antioxidant properties of these extracts.

Due to the presence of various biologically active compounds like isoimpinellin, casimiroin, skimmianine, 1-methyl-2-phenyl-4-quinol, edulein, and scopoletinmethyl ether [55], as well as flavones, the strongest antioxidant [20], treatment by C. edulis and *G. pentaphylla* displayed several activities. Furthermore, the glucoside casimirosine was present in the seed extract and other substances, including coumarins, flavonoids, and limonoids, which are known to have anti-inflammatory actions, were also discovered [23]. Therefore, various biologically active substances that showed antioxidant and anti-inflammatory activities may be responsible for alleviating neurotoxicity induced in mice.

## 5 Conclusion

In conclusion, the current study demonstrated that intoxication by AgNPs in murine model could promote apoptosis of nerve tissue through pro-apoptotic Bax mRNA overexpression and reduction of anti-apoptotic Bcl-2 mRNA gene expression in nanoparticle size-dependent manner in addition to a notable elevation of caspace-3 as well as P53 apoptotic biomarker. Furthermore, histopathological examination confirmed all the obtained results. However, treatment by natural products extracts investigated a significant modulation of all biochemical, oxidative stress as well as molecular parameters.

## Declaration of Competing Interest

The authors declare that they have no known competing financial interests or personal relationships that could have appeared to influence the work reported in this paper.

## Data Availability

No data was used for the research described in the article.
